# Pulmonary vascular permeability changes in an ovine model of methicillin-resistant *Staphylococcus aureus *sepsis

**DOI:** 10.1186/cc7720

**Published:** 2009-02-17

**Authors:** Collette C Jonkam, Kamna Bansal, Daniel L Traber, Atsumori Hamahata, Marc O Maybauer, Dirk M Maybauer, Robert A Cox, Matthias Lange, Rhykka L Connelly, Lillian D Traber, Clarisse D Djukom, John R Salsbury, David N Herndon, Perenlei Enkhbaatar

**Affiliations:** 1 Department of Anesthesiology, The University of Texas Medical Branch and Shriners Hospital for Children, 601 Harborside Drive, Galveston, TX 77555-1102, USA; 2 Department of Pathology, The University of Texas Medical Branch and Shriners Hospital for Children, 301 University Blvd, Galveston, TX 77555, USA; 3 Department of Surgery, The University of Texas Medical Branch and Shriners Hospital for Children, 301 University Blvd, Galveston, TX 77555, USA

## Abstract

**Introduction:**

Endothelial dysfunction is a hallmark of sepsis, associated with lung transvascular fluid flux and pulmonary dysfunction in septic patients. We tested the hypothesis that methicillin-resistant *Staphylococcus aureus *(MRSA) sepsis following smoke inhalation increases pulmonary transvascular fluid flux via excessive nitric oxide (NO) production.

**Methods:**

Ewes were chronically instrumented, and randomised into either a control or MRSA sepsis (MRSA and smoke inhalation) group.

**Results:**

Pulmonary function remained stable in the control group, whereas the MRSA sepsis group developed impaired gas exchange and significantly increased lung lymph flow, permeability index and bloodless wet-to-dry weight-ratio (W/D ratio). The plasma nitrate/nitrite (NOx) levels, lung inducible nitric oxide synthases (iNOS) and endothelial nitric oxide synthases (eNOS), vascular endothelial growth factor (VEGF) protein expressions and poly-(ADP)-ribose (PAR) were significantly increased by MRSA challenge.

**Conclusions:**

These results provide evidence that excessive NO production may mediate pulmonary vascular hyperpermeability in MRSA sepsis via up regulation of reactive radicals and VEGF.

## Introduction

Despite advancements in the treatment of sepsis, its sequelae remain associated with increased risk of death among patients in intensive care units (ICU) [1]. From 1979 to 2000, the incidence of sepsis in the USA increased by 13.7%, and the number of sepsis-related in-hospital deaths rose from 43,579 in 1979 to 120,491 in 2000, with Gram-positive bacteria being increasingly recognised as the most common pathogens (52.1% versus 37.6% Gram negative) [2]. Pneumonia is one of the dominant causes of sepsis. Smoke inhalation injury is frequently complicated by pneumonia [3,4]. The mortality in fire victims increases by a maximum of 20% when associated with smoke inhalation injury alone, by 40% with pneumonia alone, but concomitantly they increase the mortality by up to 60% [4].

Methicillin-resistant *Staphylococcus aureus *(MRSA) is one of the leading causes of nosocomial infections in burn patients [5]. Wang and colleagues [6] reported an increased number of patients with community-acquired MRSA bacteraemia and showed a close association with necrotizing pneumonia. *Staphylococcus aureus *has been reported to be a predominant cause (38%) of ventilator-associated pneumonia (VAP) in surgical ICUs [7]. MRSA-induced VAP causes a significantly higher rate of bacteraemia and septic shock than VAP due to methicillin-sensitive *S. aureus *[8].

The endothelium serves as a semi-selective barrier to solutes and fluid. Disruption of this barrier function as seen during inflammatory processes leads to microvascular hyperpermeability [9]. In our previous study, we showed that MRSA pneumonia and sepsis leads to fluid accumulation and lung oedema formation [10]. A positive fluid balance has been shown to be an important determinant of poor outcome in patients with lung oedema [11].

Various pathogens are known to activate different pathways in different animal models, leading to important distinctions in the host response [10,12]. Previously, we compared the pathophysiological changes of sepsis induced by *Pseudomonas aeruginosa *with those seen in MRSA sepsis and reported a significantly higher fluid accumulation and plasma nitrate/nitrite (NOx) levels in MRSA sepsis [13]. The objective of the present study was to examine the molecular and physiological aspects associated with pulmonary vascular permeability changes in MRSA pneumonia and sepsis using a modified version of our established model. This modified ovine model of MRSA sepsis provides a clinically relevant approach for future studies focusing on microvascular permeability changes in MRSA-induced sepsis.

## Materials and methods

The Institutional Animal Care and Use Committee of the University of Texas Medical Branch approved the experimental protocol for this study. All animals were handled according to guidelines established by the American Physiological Society and the National Institutes of Health (NIH). The Association of the Assessment and the Accreditation of Laboratory Animal Care accredits the Investigational-ICU at University of Texas Medical Branch, where the experiments were performed.

### Surgical preparation

Sheep (weighing 30 to 40 kg) were surgically prepared and chronically instrumented for haemodynamic monitoring as previously described [13,14]. Briefly, a right femoral artery catheter (18-GA, 36 inches; Parke-Davis, Sandy, UT, USA), a left atrial catheter (0.062 inch inner diameter (ID), 0.125 inch outer diameter (OD); Dow Corning, Midland, MI, USA) and a Swan-Ganz thermal dilution catheter (REF 131F7; Edwards Lifesciences LLC, Irvine, CA, USA) were placed. For the evaluation of pulmonary permeability, an incision was made on the right sixth intercostal space, and the efferent lymphatic vessel of the caudal mediastinal lymph node was cannulated with medical-grade tubing (Silastic catheter 0.025 inches ID, 0.047 inches OD; Dow Corning, Midland, MI, USA), by a modified technique of Staub and colleagues [15], also described by Traber and colleagues [16]. The distal end of the caudal mediastinal lymph node was ligated and the borders of the diaphragm and posterior right hemithorax were cauterised to eliminate contamination of the caudal mediastinal lymph node by systemic afferent lymphatics.

### Experimental protocol

Animals were allowed a seven-day recovery period after the operative procedure. After collecting baseline data, sheep were randomised to either a control group (n = 6) or a MRSA sepsis group (n = 8). Thereafter, a tracheostomy was performed under ketamine anaesthesia and a cuffed tracheostomy tube (10 mm diameter; Shiley, Irvine, CA, USA) was inserted.

Anaesthesia was continued with halothane and a Foley urinary retention catheter (C.R. Bard, Inc., Covington, GA., USA) was inserted in the bladder to precisely measure fluid balance. All animals were adequately resuscitated with lactated Ringer's solution, delivered initially at a rate of 3 mL/kg/hour and adjusted throughout the experimental period to prevent haemoconcentration. During the experiment, animals were allowed free access to food but not to water in order to adequately monitor fluid balance.

The MRSA sepsis animals were subjected to smoke inhalation injury according to an established protocol [10,14,17]. Briefly, 4 sets of 12 breaths (total 48 breaths) of cotton smoke were insufflated into the lungs using a modified bee smoker filled with about 50 g of burning cotton toweling. After each set of smoke inhalation, arterial carboxyhaemoglobin levels were measured as an index of lung injury. The control animals received 48 breaths of room air through the bee smoker.

Following smoke inhalation, 2.5 × 10^11 ^colony forming units (cfu) of live MRSA (strain AW6), a bloodstream isolate [18], was suspended in 30 mL of saline. Using a bronchoscope (model FB-19H; Pentax, Japan), the solution was injected into the right lower, right middle and left lower lobes. Anaesthesia was then discontinued and animals were studied in the awake state for 24 hours.

All sheep were mechanically ventilated (Servo 300; Siemens, Sweden) in a volume-controlled mode with positive end-expiratory pressure set at 5 cmH_2_O, tidal volume maintained at 15 mL/kg and a respiratory rate of 20 breaths per minute. The breath rate was periodically adjusted to maintain arterial carbon dioxide tension close to baseline values. One hundred percent oxygen was delivered in the first three hours after injury to accelerate the dissociation of carbon monoxide from haemoglobin. The fraction of inspiratory oxygen was periodically adjusted to maintain the arterial oxygen tension above 95 mmHg.

At the end of the experiment, sheep were euthanased by injection of 60 mL of saturated potassium chloride into the left atrium under deep anaesthesia with ketamine (15 mg/kg). Tissue samples were harvested, snap frozen in liquid nitrogen and stored at -80°C for later analysis.

### Measured variables

Catheters were connected to pressure transducers (REF PXMK 1590; Edwards Lifesciences LLC, Irving, CA, USA) with continuous flushing devices. The transducers were connected to haemodynamic monitors (model IntelliVue MP50, Philips, Boeblingen, Germany) used to measure central venous pressure (CVP), mean pulmonary artery pressure (MPAP), pulmonary capillary wedge pressure (PCWP), left atrial pressure, cardiac output and mean arterial pressure as previously described [19]. Cardiac index and pulmonary shunt fraction (Qs/Qt) were calculated using standard formula. Pulmonary capillary pressure (P_c_) was calculated using the formula: P_c _= (0.4 × MPAP) + (0.6 × PCWP). Arterial and venous blood gases were measured with a blood gas analyser (model GEM Premier 3000, Instrumentation Laboratory, Lexington, MA, USA). Lung lymph flow (Q_L_) was measured using graduated test tubes. Lung lymph protein (P_L_) and plasma protein (P_P_) concentrations were measured using a refractometer (National Instrument, Baltimore, MD, USA). In order to estimate the pulmonary microvascular permeability to protein, total lung protein leak per hour (P_L-tot_) was determined by multiplying the Q_L _by the P_L_. Lung lymph oncotic pressures (π_L_) and plasma oncotic pressures (π_P_) were determined through a semi-permeable membrane in a colloid osmometer (Model 4420; Wescor, Logan, UT, USA). Lung permeability index (PI_L_) was calculated using the formula: (π_L_/π_P_) × Q_L_. Fluid input and urine output were recorded every three hours and net fluid balance was derived by subtracting output from input.

### Plasma nitrate/nitrite level

Plasma nitric oxide (NO) levels were evaluated by measuring the intermediate and end products, that is NOx, using Cayman nitrate/nitrite colorimetric assay kit (Cayman Chemicals, Ann Arbor, MI, USA).

### Lung bloodless wet-to-dry weight ratio

The bloodless wet-to-dry weight (W/D) ratio, an index of lung oedema, was determined using the lower half of the right lung as previously reported [10,20].

### Lung tissue immunoblotting analyses

Vascular endothelial growth factor (VEGF), lung inducible nitric oxide synthases (iNOS) and endothelial nitric oxide synthases (eNOS), 3-nitrotyrosine (3-NT) protein and PAR expressions were measured using Western blot and quantified using NIH IMAGE J scanning densitometry [17,21].

### Lung histology

Lung tissue samples were inflated with 10% formalin, embedded in paraffin, sectioned into 6 μm pieces, stained with H&E and analysed by a pathologist as previously described [22,23].

### Statistical analysis

Statistical analysis was performed using analysis of variance and bonferroni *post hoc* test or unpaired t-test. Results are presented as mean ± standard error of the mean and a p < 0.05 was considered statistically significant.

## Results

**Figure 1:**
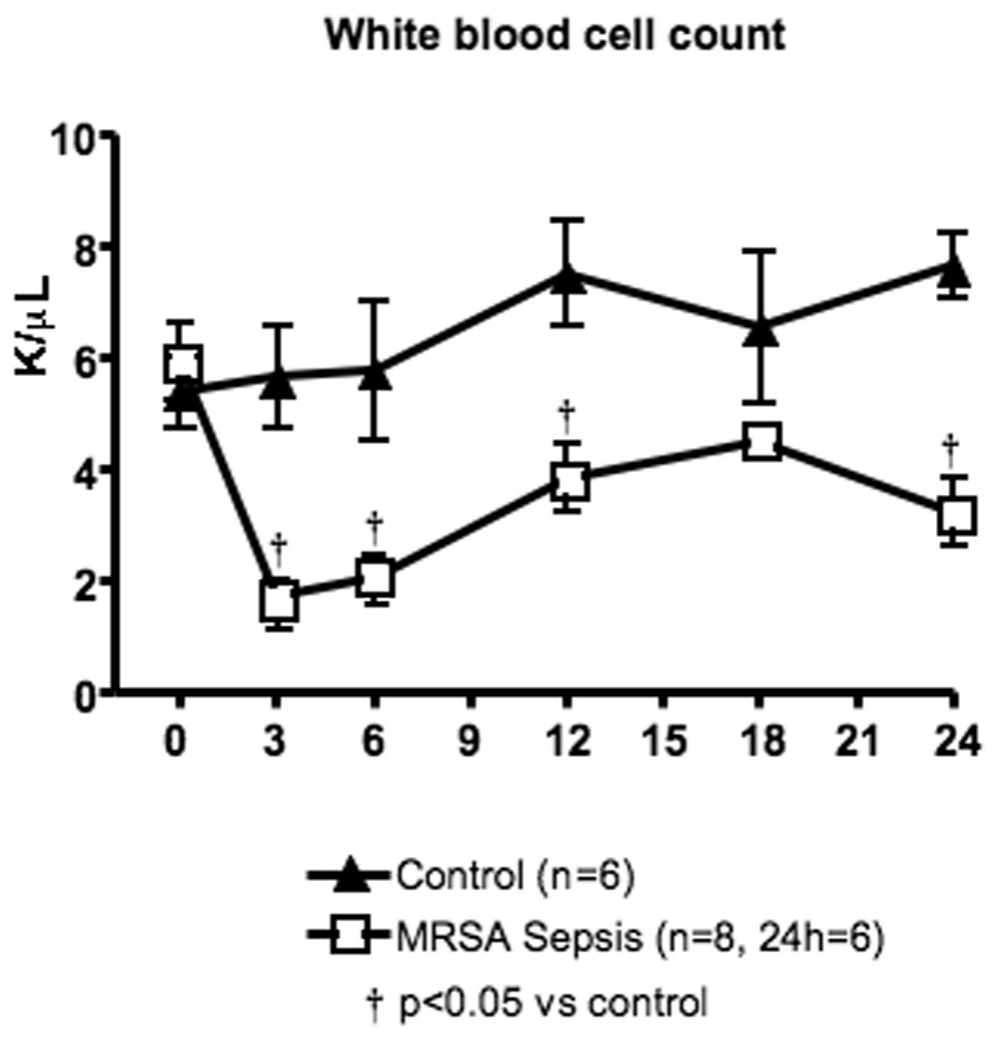
White blood cell count in control and methicillin-resistant *Staphylococcus aureus *(MRSA) sepsis groups. Data are expressed as mean ± standard error of the mean. † p < 0.05 versus control.

All six sheep in the control group survived the entire 24-hour experimental period, whereas only six of the eight sheep in the MRSA sepsis group survived 24 hours. Six of the eight animals (75%) in the MRSA sepsis group had positive blood cultures, indicating bacteraemia. The body temperature increased significantly between 3 and 12 hours (Table 1), and the white blood cell count decreased significantly in the sepsis group compared with the controls (Figure 1). Tissues were not harvested from the non-survivors.

**Table 1:** Measurements at intervals after injury

	Time after injury (hours)					
	
	BL	3	6	12	18	24
	
**Body temperature (°C)**						
Control	39.1 ± 0.16	39.6 ± 0.19	39.5 ± 0.12	39.5 ± 0.11	39.7 ± 0.27	39.9 ± 0.17
MRSA sepsis	39.2 ± 0.11	40.9 ± 0.13†	41.0 ± 0.08†	40.3 ± 0.11†	40.1 ± 0.22	40.5 ± 0.29
**CVP (mmHg)**						
Control	7.2 ± 0.8	10.8 ± 0.5	11.5 ± 1.1	10.2 ± 1.5	9.5 ± 0.6	8.8 ± 0.6
MRSA sepsis	5.5 ± 0.9	7.6 ± 1.4	8.0 ± 1.5	11.1 ± 1.2	16.7 ± 2.3†	14.5 ± 2.4
**MPAP (mmHg)**						
Control	20.2 ± 0.7	25.3 ± 1.4	27.0 ± 0.9	27.0 ± 2.0	26.7 ± 1.9	25.8 ± 1.8
MRSA sepsis	19.6 ± 0.4	23.4 ± 0.7	28.4 ± 1.6	30.0 ± 1.5	34.5 ± 1.4†	32.8 ± 2.1†
**Pc (mmHg)**						
Control	12.9 ± 0.9	17.1 ± 0.8	17.8 ± 0.5	17.4 ± 1.6	17.4 ± 1.3	16.7 ± 1.2
MRSA sepsis	12.4 ± 0.5	13.5 ± 0.6	15.8 ± 1.0	19.6 ± 0.8	23.5 ± 1.3†	22.4 ± 1.9†
**P_L-tot _(mg/hour)**						
Control	147 ± 30	117 ± 27	147 ± 30	211 ± 65	228 ± 78	267 ± 74
MRSA sepsis	122 ± 38	539 ± 115†	728 ± 124†	636 ± 140†	610 ± 105†	611 ± 198
**CI (mL/min^-1^/m^-2^)**						
Control	5.8 ± 0.2	5.5 ± 0.2	6.0 ± 0.8	5.3 ± 0.6	5.3 ± 0.4	4.9 ± 0.4
MRSA sepsis	6.2 ± 0.3	7.0 ± 0.4	6.7 ± 0.4	8.1 ± 0.5†	10.2 ± 0.9†	9.2 ± 0.8†
**HR (bpm)**						
Control	94 ± 1.9	110 ± 3	116 ± 4.9	107 ± 6.2	104 ± 6.2	103 ± 5.9
MRSA sepsis	95 ± 4.1	155 ± 11.5†	162 ± 7†	167 ± 7.4†	156 ± 7.6†	142 ± 15†
**PaO_2_/FiO_2 _ratio**						
Control	509 ± 15	482 ± 35	519 ± 16	501 ± 20	532 ± 32	521 ± 12
MRSA sepsis	504 ± 7	287 ± 24†	304 ± 27†	215 ± 27†	156 ± 23†	123 ± 26†
**Qs/Qt**						
Control	0.15 ± 0.01	0.19 ± 0.01	0.17 ± 0.02	0.2 ± 0.03	0.19 ± 0.02	0.18 ± 0.01
MRSA Sepsis	0.16 ± 0.01	0.31 ± 0.01†	0.28 ± 0.02†	0.38 ± 0.03†	0.44 ± 0.03†	0.49 ± 0.05†

All data are shown in control and methicillin-resistant *Staphylococcus aureus *(MRSA) sepsis groups. Data are expressed as mean ± standard error of the mean. † p < 0.05 versus control.

BL = baseline; CI = cardiac index; CVP = central venous pressure; HR = heart rate; FiO_2 _= fraction of inspiratory oxygen; MPAP = mean pulmonary artery pressure; PaO_2 _= partial arterial pressure of oxygen; Pc = pulmonary capillary pressure; P_L-tot _= total lung lymph protein; Qs/Qt = pulmonary shunt fraction.

There were no significant changes in cardiopulmonary function in the control animals. The injured animals developed significantly elevated CVP, MPAP, P_c_, cardiac index and heart rate compared with controls (Table 1). These changes were associated with severe pulmonary dysfunction in the MRSA sepsis group, evidenced by significantly decreased partial arterial pressure of oxygen (PaO_2_)/fraction of inspired oxygen (FiO_2_) ratio and increased Qs/Qt (Table 1). The MRSA group showed a significantly higher bronchial obstruction score (20.6 ± 2.8% airway obstruction) compared with the controls (2.5 ± 0.8%; p < 0.0001).

**Figure 2:**
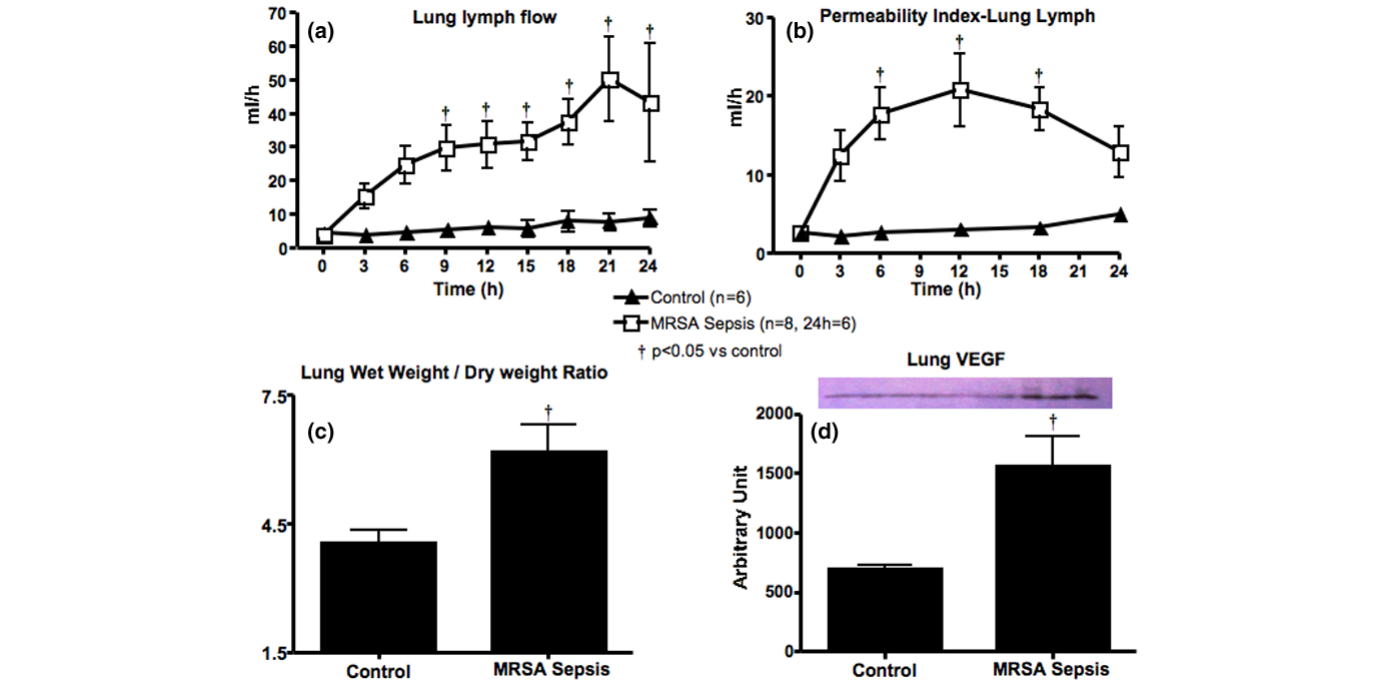
Changes in lung permeability and VEGF measured in control and methicillin-resistant *Staphylococcus aureus *(MRSA) sepsis groups. **(a)** Lung lymph flow, **(b) **lung permeability index, **(c)** lung wet/dry weight-ratio and **(d)** vascular endothelial growth factor (VEGF). Data are expressed as mean ± standard error of the mean. † p < 0.05 versus control.

The Q_L_, PI_L_, P_L-tot_, lung W/D-ratio and VEGF protein expression increased significantly in the MRSA sepsis group compared with the control (Figure 2 and Table 1).

**Figure 3:**
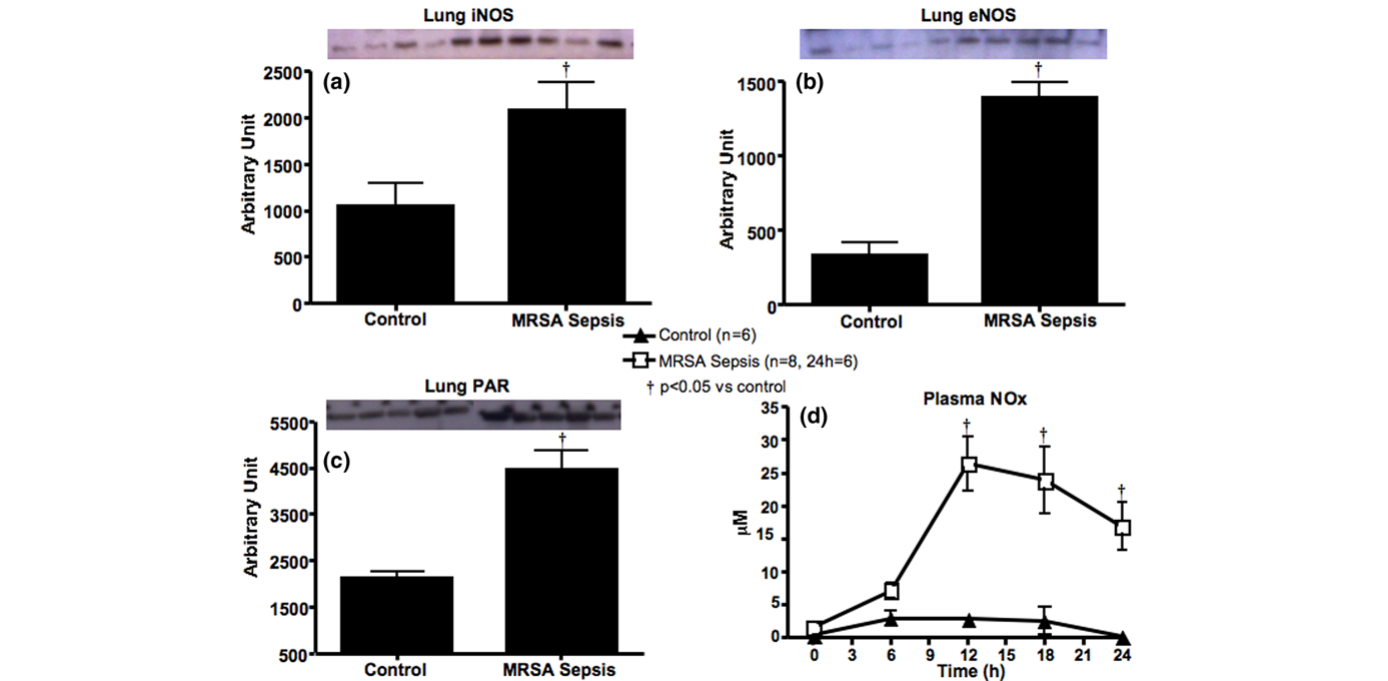
Excessive nitric oxide production and PAR measured in control and methicillin-resistant *Staphylococcus aureus *(MRSA) sepsis groups. **(a) **Lung inducible nitric oxide synthase (iNOS) and **(b)** endothelial nitric oxide synthase (eNOS); **(c)** lung poly ADP ribose (PAR) and **(d)** plasma nitrite/nitrate (NOx). Data are expressed as mean ± standard error of the mean. † p < 0.05 versus control.

These permeability changes were accompanied by significantly increased plasma NOx levels, lung iNOS and eNOS protein expressions in the septic animals compared with the control group (Figure 3). Lung PAR expression, an index of poly-ADP ribose polymerase (PARP) activity, was also significantly increased in the septic animals compared with the control group (Figure 3). Lung 3-NT protein expression, an index of tissue peroxynitrite (ONOO^-^) formation, showed an increasing tendency after injury (control: 19032.4 ± 1207.6; MRSA sepsis: 22264.2 ± 1097.3; p = 0.08).

The 95% confidence interval, mean difference (interpretable as the size of the effect, if the outcome is measured on a familiar scale) and p values of lung lymph flow and permeability index, plasma NOx, lung iNOS, eNOS, PAR, VEGF and W/D ratio are shown in Table 2.

**Table 2:** Confidence interval, mean difference and p values of data

**Lung lymph flow**	**95% CI**	**Mean difference**	**p value**
BL = 0 hours	-24.77 to 23.68	-0.5458	> 0.05
3 hours	-12.68 to 35.78	11.55	> 0.05
6 hours	-3.977 to 44.48	20.25	> 0.05
9 hours	0.1814 to 48.64	24.41	< 0.05
12 hours	0.2981 to 48.75	24.53	< 0.05
15 hours	0.9306 to 50.85	25.89	< 0.05
18 hours	4.697 to 54.61	29.65	< 0.01
21 hours	16.22 to 68.75	42.49	< 0.001
24 hours	6.188 to 62.93	34.56	< 0.01

**Permeability index-lung lymph**			

BL = 0 hours	-10.13 to 10.09	-0.01615	> 0.05
3 hours	-0.2701 to 20.64	10.19	> 0.05
6 hours	5.077 to 25.30	15.19	< 0.01
12 hours	7.769 to 27.99	17.88	< 0.001
18 hours	4.669 to 25.58	15.12	< 0.01
24 hours	-5.223 to 21.15	7.964	> 0.05

**Plasma NOx**			

BL = 0 hours	-13.91 to 16.26	1.171	> 0.05
6 hours	-7.868 to 16.32	4.225	> 0.05
12 hours	10.52 to 36.41	23.46	< 0.001
18 hours	7.066 to 35.57	21.32	< 0.01
24 hours	3.710 to 29.60	16.66	< 0.01
**Lung iNOS**	1945 to 132.6	1039	0.0296
**Lung eNOS**	1381 to 731.0	1056	< 0.0001
**Lung PAR**	3401 to 1298	2349	0.0009
**Lung VEGF**	1477 to 248.4	862.6	0.0119
**Lung wet/dry ratio**	3.846 to 0.4543	2.150	0.0176

BL = baseline; CI = confidence interval; eNOS = endothelial nitric oxide synthase; iNOS = inducible nitric oxide synthase; NOx = nitrate/nitrite; PAR = poly-(ADP)-ribose; VEGF = vascular endothelial growth factor.

## Discussion

Sepsis caused by MRSA is most often associated with severe outcomes. Previous studies indicate that MRSA pneumonia and sepsis induces significantly higher plasma NOx levels and net fluid accumulation compared with *P. aeruginosa *sepsis [10,14,24]. The present study focuses on pulmonary vascular permeability changes in MRSA sepsis. Injured animals developed hyperdynamic sepsis, evidenced by increased cardiac index, heart rate and body temperature, decreased leucocyte count and the presence of bacteraemia. These changes in the MRSA sepsis group were associated with bronchial obstruction and pulmonary dysfunction. The increased Q_L _and PI_L _observed in the MRSA sepsis group signify increased pulmonary transvascular fluid flux. According to the Starling equation J_v _= K_f_((P_c_-P_i_) - ∂(π_c _- π_i_)), transvascular fluid filtration is determined by the capillary and interstitial hydrostatic (P_c_-P_i_) and colloid osmotic pressures (π_c_-π_i_) where ∂ is the reflection coefficient [25,26]. P_L-tot _increased in the sepsis group, indicating increased permeability to protein in the pulmonary vasculature.

Previously we showed that sheep subjected to smoke inhalation injury alone developed neither a decrease in plasma protein concentration and oncotic pressure nor an increase in fluid accumulation compared with uninjured controls [10]. This suggests that smoke inhalation injury alone does not induce vascular hyperpermeability to protein, but rather that the increased protein leakage is due to MRSA sepsis. The current study showed increased P_c _consistent with the findings of Isago and colleagues [27], that showed that increased P_c _contributes to lung oedema formation after acute lung injury. When the capacity of the lymphatic system is exceeded, fluid tends to accumulate in the interstitial space, leading to oedema formation. The increased lung W/D-ratio and Q_L _seen in the sepsis group reflects this theory.

Excessive NO is implicated in many pathophysiological changes of sepsis. Plasma NOx levels, lung iNOS and eNOS protein expressions significantly increased after injury. This is in agreement with other studies, which suggest that both iNOS-derived and eNOS-derived NO plays a pivotal role in vascular hyperpermeability during sepsis [28-30]. Although iNOS is recognised as the dominant enzyme responsible for the sepsis-related cardiovascular derangements, constitutive NOS (neuronal NOS and eNOS) have also been reported to play a major role in sepsis [31-33]. MRSA could cause severe pulmonary vascular hyperpermeability via upregulation of both iNOS and eNOS.

Situated in close proximity to one another, NO reacts with superoxide (O_2_^-^) to form ONOO^-^. ONOO^-^, a potent protein nitrating species, is known to cause DNA single strand breakage [34], vascular contractile, and endothelial dysfunction [35,36]. Our group previously reported that the ONOO^- ^decomposition catalyst WW-85 decreased lung transvascular fluid flux in sheep with IL-2-induced increase in pulmonary vascular permeability [37]. Although lung 3-NT expression was not significantly higher in the MRSA group, we speculate that significance may have been reached at an earlier time point, especially because plasma NOx levels showed an early increase followed by a decreasing tendency at 24 hours. ONOO^-^-induced breakage of single-strand DNA triggers excessive PARP activation that in turn causes cellular ATP depletion, tissue damage and cell death [38,39]. Lung PAR expression was significantly upregulated, suggesting that excessive NO levels following MRSA sepsis could cause increased pulmonary vascular leakage directly or through ONOO^- ^formation with subsequent PARP over activation.

The endothelium is known to play a key role in the modulation of vascular permeability [40-42]. This study is in line with our previous study [21], which showed that VEGF, a known potent vascular permeability factor [43], is overexpressed in lung tissue after injury, suggesting that MRSA may cause disruption of the endothelial integrity, leading to pulmonary vascular hyperpermeability and lung oedema. Whether NO upregulates VEGF production or vice-versa is still controversially discussed. Kroll and colleagues [44] previously reported that the activation of VEGF receptor-2 upregulates iNOS and eNOS production. However, Heo and colleagues[45] have shown that NOS inhibition using L-NAME reduced lipopolysaccharide-induced NO and VEGF production in human aortic smooth muscle cells. Furthermore, L-NAME has been reported to inhibit iNOS-derived and eNOS-derived NO-induced VEGF up regulation in rat colon [46], supporting the theory that excessive NO may stimulate VEGF expression.

A limitation of this study that we would like to acknowledge is the fact that only female sheep were used. We cannot guarantee that male sheep would show exactly the same response. However, we believe that the molecular and pathophysiological changes in male sheep subjected to the same injury would show the same trend as those seen in the current study. Secondly, the response seen in the lung tissue could be earlier than would be the case in humans, because both smoke inhalation and bacteria were introduced directly into the lungs.

## Conclusions

The current study provides evidence that the severe transvascular fluid flux in the pulmonary system induced by MRSA pneumonia and sepsis may be mediated by iNOS-generated and eNOS-generated excessive NO via augmentation of reactive nitrogen species, PARP and VEGF. We believe that this modified MRSA pneumonia and sepsis model might provide a clinically relevant and useful new approach for studying new therapeutic strategies on endothelial dysfunction and its outcome. It would be of interest to investigate the time course (early versus late onset) of the expression of different NOS isoforms and VEGF after MRSA pneumonia and sepsis, and to evaluate the role of specific NOS inhibitors on MRSA sepsis-induced vascular hyperpermeability.

## Key messages

○ MRSA sepsis causes severe vascular leakage in the pulmonary system.

○ Excessive NO production mediates pulmonary vascular hyperpermeability via upregulation of VEGF, OONO^- ^and PARP activity.

## Abbreviations

3-NT: 3-nitrotyrosine; CFU: colony forming units; CVP: central venous pressure; eNOS: endothelial nitric oxide synthase; FiO_2_: fraction of inspiratory oxygen; H&E: haematoxylin & eosin; ICU: intensive care unit; ID: inner diameter; IL: interleukin; iNOS: inducible nitric oxide synthase; MPAP: mean pulmonary artery pressure; MRSA: methicillin-resistant *Staphylococcus aureus*; NIH: National Institutes of Health; NO: nitric oxide; NOx: nitrate/nitrite; O_2_^-^: superoxide; OD: outer diameter; ONOO^-^: peroxynitrite; PaO_2_: partial arterial pressure of oxygen; PAR: poly-(ADP)-ribose; PARP: poly-(ADP)-ribose polymerase; P_c_: pulmonary capillary pressure; PCWP: pulmonary capillary wedge pressure; PI_L_: lung permeability index; P_L_: lung lymph protein; P_L-tot_: total lung lymph protein content; P_P_: plasma protein; π_L_: lung lymph oncotic pressure; π_P_: plasma oncotic pressure; Q_L_: lung lymph flow; Qs/Qt: pulmonary shunt fraction; VAP: ventilator associated pneumonia; VEGF: vascular endothelial growth factor; W/D ratio: wet-to-dry-weight ratio.

## Competing interests

The authors declare that they have no competing interests.

## Authors' contributions

CCJ designed and carried out the experiments, and analysed and interpreted the data. KB contributed in the performance, analysis and interpretation of immunoblotting assays. DLT contributed with grant support, study design and interpretation of the data. AH performed the complicated surgeries and contributed in the analysis of the data. MOM and DMM drafted the manuscript and revised it critically for important intellectual content. RAC designed, performed and analysed the lung histology data. ML collected, analysed and interpreted some of the data. RLC designed, analysed and interpreted the immunoblotting assays. LDT performed the complicated surgeries. CDD collected and analysed the data. JRS contributed in the performance of the surgeries and the experiments. DNH contributed in designing the experiment. PE contributed with grant support, designed the experiment and interpreted the data. CCJ, DLT, MOM, DMM and PE drafted the manuscript. All authors read and approved the final manuscript.
